# Genome Assembly and Annotation of the Alfalfa Cyst Nematode (*Heterodera medicaginis*)

**DOI:** 10.2478/jofnem-2025-0047

**Published:** 2025-10-05

**Authors:** Olga A. Postnikova, Catherine Wram, Sam Grinstead, Paulo Vieira, Lev G. Nemchinov

**Affiliations:** USDA/ARS/BARC/Animal Biosciences and Biotechnology Laboratory, Beltsville, MD; USDA-ARS, Mycology and Nematology Genetic Diversity and Biology Laboratory, Beltsville, MD; USDA-ARS, Molecular Plant Pathology Laboratory, Beltsville, MD

**Keywords:** alfalfa cyst nematode, genome assembly, *Heterodera medicaginis*, Oxford Nanopore sequencing

## Abstract

The alfalfa cyst nematode (*Heterodera medicaginis*) is an invasive and pathogenic species parasitizing the agriculturally important forage crop alfalfa (*Medicago sativa* L.). To date, no genome assembly of *H. medicaginis* has been reported in the literature. In this study, for the first time, we assembled a draft genome of this species. This research is important as it provides genomic insights into a damaging pest recently discovered in the United States that affects the third most valuable crop in the country.

Cyst nematodes (*Heterodera* and *Globodera* spp.) are widely recognized as the second most economically significant group of plant-parasitic nematodes, following root-knot nematodes, due to their widespread distribution and the substantial yield losses they cause in a variety of important food crops (Jones et al., 2013). These root sedentary endoparasites are obligate biotrophs that depend entirely on a living host for their survival and reproduction. Cyst nematodes secrete dozens of effectors produced in esophageal glands. These effectors play crucial roles in manipulating host cellular functions, including suppression of defense responses, modulation of host gene expression and reprogramming cellular development (Bali and Gleason, 2024; Molloy et al., 2024). While some effectors are conserved among various plant-parasitic nematodes, others exhibit a high degree of specificity, being restricted to cyst nematodes or even species-specific (Pokhare et al., 2020; Molloy et al., 2024).

The alfalfa cyst nematode (*Heterodera medicaginis*) is an invasive, pathogenic species that parasitizing the agriculturally important forage crop alfalfa (*Medicago sativa* L.). It was first discovered in the Poltava region, Ukraine (Steinberg, 1932), established as a new species in 1971 (Kirjanova and Krall, 1971), and redescribed in 1982 (Gerber and Maas, 1982). It is distributed in Europe (Russia and Ukraine) and Asia (Kazakhstan and Uzbekistan) (Subbotin et al., 2010). In the United States, it was first detected in soil samples from Kansas and Montana (Powers et al., 2019) and later in an alfalfa field in Utah (Handoo et al., 2020). *H. medicaginis* can cause severe yield losses in alfalfa, reducing green matter by up to 46% (Subbotin et al., 2010). Its negative impact is particularly pronounced in young alfalfa plants under arid conditions (Subbotin et al., 2010).

To date, no genome assembly of *H. medicaginis* is publicly available. In contrast, comprehensive genomic datasets have been generated for closely related cyst nematodes, including the soybean cyst nematode, *H. glycines* (Masonbrick et al., 2019) and the sugar beet cyst nematode, *H. schachtii* (Siddique et al., 2022; Molloy et al., 2024). Given their agricultural significance and their host-specific adaptations, comparative genomic analysis can offer valuable insights into the genetic basis of their host range, parasitism strategies, and evolutionary divergence within the *Heterodera* genus and beyond. Using long-read DNA sequences generated by Oxford Nanopore Technologies (ONT), we have assembled the first draft genome of *H. medicaginis*.

To obtain fresh nematodes for DNA extraction, one liter of naturally infested soil with ~1,000 encysted eggs of *H. medicaginis*/100 cc was provided by Dr. Timothy Todd, Kansas State University. Prior to establishing *H. medicaginis* in culture, a 25 cc subsample of soil samples was used for cyst extraction by sieving a soil–water mixture through a 250 µm sieve. The recovered cysts were handpicked and examined under an Olympus BX53 light microscope for morphological identification. The infested soil was initially used to grow alfalfa seedlings of cultivars Samarac and Mesa-Sirsa for ~60 d in greenhouse conditions with 16 hr light/8 hr dark. Cysts were then extracted from the soil as described above, surface-sterilized in sterile water containing 50 mg/l carbenicillin and 50 mg/l kanamycin, and placed in 3 mM ZNCl^2^ to initiate hatching. The resulting J2s were collected and used for the infection of 10 new alfalfa plants of both cvs. Samarac and Mesa-Sirsa are growing in a sterile soil mixture. After ~60 d, plants were destructively harvested and cysts collected from the soil. Characteristic *H. medicaginis* cysts with eggs were observed (Fig. S1 in Supplementary Material). No other cyst nematodes are known to infect alfalfa. To obtain eggs from cysts, 100 cysts were handpicked, surface-sterilized, and cut with a sterile blade to release eggs. Roughly, 750 eggs were collected in a 2 ml tube and frozen in liquid nitrogen before DNA extraction. To lyse the eggs, a QIA TissueLyser LT (Qiagen, Hilden, Germany) with 5 mm stainless steel beads was used to bead-beat the sample at 50 oscillations for 60 sec.

DNA was then extracted using a Promega HMW DNA extraction kit (Promega, Madison, WI) following the manufacturer’s instructions. DNA was amplified using QIA REPLI-G Mini (Qiagen) whole genome amplification kit. To prepare DNA for sequencing, libraries were generated using a Ligation Sequencing kit (Oxford Nanopore Technologies, Oxford, UK) according to the manufacturer’s instructions. Two DNA libraries were sequenced on MinION (Oxford Nanopore Technologies) with FLO-MIN106D flow cell for 24 hr each generating 307,627 and 379,309 reads, respectively. The first library had 2.15 Gbp with an average read length of 6,990 bp and a maximum length of 115,890 bp. The second library consisted of 2.20 Gbp with an average read length of 5,793 bp and a maximum read length of 101,226 bp.

Nanopore sequences were base-called with Dorado using the sup model (https://github.com/nanoporetech/dorado). The data were then pooled together and quality trimmed with porechop (Wick et al., 2017). The pore-chopped data were assembled with both the wtdbg2 (RedBean) assembler and Canu assembler using default parameters (Koren et al., 2017; Ruan and Li, 2019). The resulting assemblies were merged with Quickmerge (Chakraborty et al., 2016) and then checked and corrected for assembly errors with the Inspector reference-free assembly evaluator (Chen et al., 2021) and the pore-chopped ONT reads. To filter the assembly for contamination, the same methodology described in Núñez-Rodríguez et al. (2024) and Wram et al. (2022) was used. Briefly, Blobtools v. 1.0 (Laetsch and Blaxter, 2017) and BLAST were used to give each contig a phylum identity based on similarity to (E-value <10e^−25^) sequences either in NCBI’s “nt” database or to a curated plant-parasitic nematode database as outlined in Núñez-Rodríguez et al. (2024). Contigs were retained if they belonged to “Nematoda” or had “nohit.” After the contigs determined to be contaminated via the Blobtools method were removed, further contamination screening was done with NCBI FCSGX (Astashyn et al., 2024). The resulting contigs or portions of contigs deemed contaminated based on results from the FCS-GX analysis were removed to generate the final assembly. Final assembly statistics were generated using QUAST (Mikheenko et al., 2018). This resulted in a 112,910,497 bp genome in 1,383 contigs with an average of 44.8× read coverage (Table 1). Three *Heterodera* genomes, *H. glycines*, *H. schachtii*, and *H. filipjevi*, were 1.40, 1.59, and 1.20× larger, respectively, and two other genomes, *H. humuli* and *H. carotae*, were 1.24 and 1.19× smaller than that of *H. medicaginis*, respectively. The N50 value for *H. medicaginis*, indicating the quality of the assembly, is 148,984 bp, whereas for other *Heterodera* species it varies from 13,935 bp to >17 Mb in the case of *H. glycines* (Table 1). The GC content for each of the genomes is rather similar, with 37% for *H. medicaginis* and 37%, 33%, 36%, 39%, and 36.7% for *H. glycines*, *H. schachtii*, *H. humuli*, *H. carotae*, and *H. filipjevi*, respectively (Table 1).

**Table 1:** Genome assembly statistics of *H. medicaginis* and other *Heterodera* species.

***Assembly statistic***	***H. medicaginis***	***H. glycines* (PRJNA381081)^a^**	***H. schachtii* (PRJNA522950)^a^**	***H. humuli* (PRJNA1048471)^a^**	***H. carotae* (PRJNA774818)^a^**	***H. filipjevi* (PRJNA1113137)^a^**
Read type	Nanopore	PacBio/Illumina	PacBio/Illumina	PacBio	Illumina	PacBio RS/Illumina
Total length	112,910,497	157,978,452	179,246,932	90,806,450	95,115,141	134,189,547
Number of contigs/Number of Scaffolds	1,383/1,383	2,121/9	1,682/395	1,487/1,487	17,835/17,835	1,208/661
Largest contig	1,183,061	23,985,585	6,046,013	568,746	113,425	30,258,874
Number of contigs (≥25,000 bp)	927	9	309	871	699	210
Number of contigs (≥50,000 bp)	655	9	269	559	103	63
GC (%)	37	37	33	36	39	36.76
N50	148,984	17,907,690	1,273,070	111,383	13,935	11,883,475
L50	220	4	40	231	1,836	4
Number of N’s per 100 kbp	0	1,062	2,756	0	0	564.5
RNA-seq evidence used for annotation	No	Yes	Yes	No	No	Yes
Coding genes	17,779	22,465	26,739	15,428	18,781	13,352
^b^CDS	20,698	23,917	32,600	16,848	20,104	13,352
Proteins	20,698	23,917	32,600	16,848	20,104	13,352
Predicted protein BUSCO analysis (Nematoda_odb10)						
^c^Complete BUSCOs (C)	1,757 (56.1%)	1,884 (60.2%)	2,080 (66.4%)	2,030 (64.9%)	1,301 (41.5%)	1,873 (59.8%)
Complete and single-copy BUSCOs (S)	1,696 (54.2%)	1,612 (51.5%)	1,675 (53.5%)	1,590 (50.8%)	1,237 (39.5%)	1,805 (57.6%)
Complete and duplicated BUSCOs (D)	61 (1.9%)	272 (8.7%)	405 (12.9%)	440 (14.1%)	64 (2%)	68 (2.2%)
Fragmented BUSCOs (F)	108 (3.4%)	47 (1.5%)	69 (2.2%)	66 (2.1%)	124 (4%)	102 (3.3%)
Missing BUSCOs (M)	1266 (40.5%)	1,200 (38.3%)	982 (31.4%)	1,035 (33%)	1,706 (54.5%)	1,156 (36.9%)
Total BUSCO groups searched	3,131	3,131	3,131	3,131	3,131	3,131
Predicted protein BUSCO analysis (Eukaryota_odb10)						
^c^Complete BUSCOs (C)	297 (77.3%)	199 (78%)	224 (87.8%)	221 (86.6%)	160 (62.8%)	205 (80.4%)
Complete and single-copy BUSCOs (S)	194 (76.1%)	176 (69%)	200 (78.4%)	174 (68.2%)	156 (61.2%)	194 (76.1%)
Complete and duplicated BUSCOs (D)	3 (1.2%)	23 (9%)	24 (9.4%)	47 (18.4%)	4 (1.6%)	11 (4.3%)
Fragmented BUSCOs (F)	20 (7.8%)	20 (7.8%)	15 (5.9%)	13 (5.1%)	52 (20.4%)	13 (5.1%)
Missing BUSCOs (M)	38 (14.9%)	36 (14.2%)	16 (6.3%)	21 (8.3%)	43 (16.8%)	37 (14.5%)
Total BUSCO groups searched	255	255	255	255	255	255

a NCBI GeneBank BioProject Accession.

b This includes multiple isoforms that result from a single coding gene.

c Of which 195 contain internal stop codons.

Genome completeness was assessed using both the Nematoda and Eukaryota standards (nematode_odb10 and eukaryota_odb10) with BUSCO v.5.7.1 software (Manni et al., 2021). The *H. medicaginis* genome was found to be comparable to the assembled genomes of other species in the genera and had complete BUSCO scores of 56.1% (nematoda_odb10) and 77.3% (eukaryote_0db10) (Table 1). These results are comparable to other available genomes of other plant-parasitic nematodes (Howe et al., 2017). The single-copy BUSCO genes in *H. medicaginis* genome comprised 54.2% (nematoda_odb10), which is higher than in all other *Heterodera* species, except *H. filipjevi* (57.6%). A visualization of the final *H. medicaginis* genome assembly is shown in Figure S2 in Supplementary Material.

Annotation of the *H. medicaginis* genome was completed in two phases. First, highly repetitive regions were identified and masked using RepeatModeler v. 2.0.2 and RepeatMasker v 4.1.0, respectively, using default parameters (Smit and Hubley, 2015a, 2015b). Then, BRAKER3 v 3.0.8 (Lomsadze et al., 2005; Stanke et al., 2006, 2008; Gotoh, 2008; Iwata and Gotoh, 2012; Buchfink et al., 2015; Hoff et al., 2016, 2019; Brůna et al., 2020, 2021) was used to annotate the *H. medicaginis* masked genome, with predicted proteins from the *H. glycines* genome (Masonbrink et al., 2019) provided as protein hints.

The number of complete coding genes in *H. medicaginis* (17,779) was also comparable with other *Heterodera* species (22,465; 26,739; 15,428; 18,781; and 13,352 in *H. glycines*, *H. schachtii*, *H. humuli*, *H. carotae*, and *H. filipjevi*, respectively) (Table 1). The number of CDS (coding DNA sequence) translating into proteins in *H. medicaginis* (20,698) was more in line with *H. carotae* (20,104) than with other *Heterodera* species (32,600; 16,848; 23,927; and 13,352 in *H. schachtii*, *H. humuli*, *H. glycines*, and *H. filipjevi*, respectively). To identify potentially secreted proteins, all amino acid sequences were checked for the presence of a signal peptide using SignalP6 fast version (Teufel et al., 2022). Of the 20,698 proteins, 2,036 contained a signal peptide sequence; these signal peptide sequences were removed from the protein sequences to identify the presence of transmembrane domains using bedtools v 2.30.0 (Quinlan and Hall, 2010) and samtools v 1.17 (Danecek et al., 2021). Once signal peptide sequences were removed from the protein sequences, transmembrane domains were detected with DeepTMHMM v 1.17 (Hallgren et al., 2022), resulting in a total of 1,703 proteins that contained a signal peptide and no transmembrane domains. To validate the presence of known nematode effector proteins, BLAST analyses (e-value 1e^−5^ and bitscore >50) were performed against the effectorome of other cyst nematodes (Masonbrick et al., 2019; Siddique et al., 2022; Molloy et al., 2024). A substantial number of candidate effectors were identified among the predicted secreted proteins of* M. medicaginis*, including several cell wall-degrading enzymes often described across multiple plant-parasitic nematodes, and an expanded family of secreted glutathione synthetases, commonly found associated with other cyst and reniform nematodes, among others (Lilley et al., 2018; Molloy et al., 2024). A comprehensive analysis of the complete effector repertoire of *H. medicaginis* will be provided in a separate study.

We used both *coxI* and *hsp90* genes, recognized markers for nematode molecular systematics (Skantar and Carta, 2004; Subbotin et al., 2025), to construct dendrograms comparing our isolate with various nematode species. The NCBI accessions are denoted for all sequences used to generate each dendrogram in Figures S3 and S4 in Supplementary Material. Sequences for each respective gene were aligned with MUSCLE integrated on CLC Genomics Workbench v 24 (Qiagen) using default parameters. Maximum likelihood trees were generated for each gene with 1000 bootstrap alignments and visualized using CLC Genomics Workbench. The groupings observed in both gene trees mirror those found by Powers et al. (2019) and Subbotin et al. (2025).

The cyst nematode *H. medicaginis* was only recently discovered in the United States (Powers et al., 2019; Handoo et al., 2020). As other phylogenetically close nematodes of the genus are of substantial economic importance in different crops (e.g., soybean and sugar beet), the availability of the genome of the alfalfa cyst nematode is critical to highlighting their potential biological and evolutionary relationships.

The genome of *H. medicaginis* can serve as a valuable resource for the community of researchers dedicated to the improvement of alfalfa and other crops. It can expedite the discovery of the *H. medicaginis* effectors and identification of other key genes involved in pathogenesis and host interaction, a better understanding of the nematode’s biology, and ultimately the development of control measures.
